# Do Adolescent Hearing Aid Users Prefer Digital Noise Reduction to Be Activated? Findings From the Laboratory and Home Environments

**DOI:** 10.1097/AUD.0000000000001794

**Published:** 2026-02-13

**Authors:** Erin M. Picou, Kjersten Branscome, Kayleigh Pletch, Lisa Standaert, Andrea H. Dunn, Johanna Nelson

**Affiliations:** 1 Department of Hearing and Speech Sciences, Vanderbilt University Medical Center, Stäfa, Switzerland; 2 Sonova, AG, Stäfa, Switzerland.

**Keywords:** Children, Field trial, Hearing aid, Noise reduction, Preference, Speech in noise

## Abstract

**Objectives:**

The purpose of this study was to evaluate the effects of an advanced digital noise reduction algorithm on measures in the laboratory (double-blind paired comparison testing, unblinded slider setting, sentence recognition performance) and during a field trial (unblinded slider setting). A secondary purpose was to evaluate participants’ ability and willingness to use a smartphone application for controlling the algorithm in the field.

**Design:**

Laboratory procedures included the evaluation of subjective ratings of listening ease using a double-blinded, paired comparisons approach while listening to speech in background noise. Participants were trained to use a smartphone application for manually controlling the advanced digital noise reduction algorithm and they indicated their preferred setting using the smartphone application, also in the laboratory. In addition, they completed double-blinded, behavioral sentence recognition in noise testing with a variety of advanced noise reduction settings. Finally, participants wore the hearing aids at home during a field trial, with instructions to use the smartphone application during the trial in noisy situations and to report on their experiences using a bespoke questionnaire upon their return to the laboratory.

**Results:**

Double-blind, paired comparison testing revealed that most participants (80%) preferred to have advanced digital noise reduction active in the noisy, reverberant laboratory. These participants were also likely to demonstrate a preference for the advanced digital noise reduction to be active during the unblinded preference task. Advanced digital noise reduction did not affect sentence recognition in noise performance. During the field trial, participants could use the smartphone application to adjust the advanced noise reduction strength in noisy situations but did not choose to do so frequently. In addition, on average, participants did not activate the advanced digital noise reduction algorithm when in self-identified difficult listening situations during the field trial.

**Conclusions:**

The results of the current study demonstrate robust subjective benefits of advanced digital noise activation in the laboratory, with no effects on speech intelligibility. In addition, participants were internally consistent in the laboratory; their self-adjusted settings were consistent with the program they preferred during the double-blind, paired comparisons testing. However, the findings with the smartphone application demonstrate that, in general, they did not activate the advanced digital noise reduction during their self-identified difficult listening situations in the field. This result could partially be explained by the limited reported use of the smartphone application during the field trial. Future study is warranted to reconcile the laboratory and field trial findings in this study. In the interim, a reasonable clinical approach with limited negative speech intelligibility consequences might be to activate advanced digital noise reduction by default and provide smartphone application access in case a patient discovers a preference for an alternative noise reduction strength.

## INTRODUCTION

Children are often in social-, vocational-, and school-based listening environments that exceed recommended noise limits (Bess et al. 1984; Shield & Dockrell 2004; American National Standards Institute 2010; Crukley et al. 2011; Spratford et al. 2019). Noise in these environments can interfere with speech understanding (Neuman et al. 2010; Valente et al. 2012), especially for children with hearing loss (Gravel et al. 1999; Crandell & Smaldino 2000; Pittman 2011b), and has been associated with increased anxiety (Standing & Stace 1980; Živojinović et al. 2023). Although hearing aids can improve audibility, children with hearing loss wearing hearing aids still require a more favorable signal to noise ratio (SNR) than do their peers with typical hearing (Gravel et al. 1999; Browning et al. 2019). Therefore, advanced hearing aid technologies that can mitigate the effects of noise are necessary to further improve listening in noise for children with hearing loss in academic, vocational, and social environments with background noise. Fortunately, several technologies are available for school-aged children.

For example, directional hearing aid microphones have reduced sensitivity to sounds arriving at the hearing aid microphones from certain azimuths—typically from the back and/or to the side of the listener (Ricketts 2000). This differential sensitivity can result in small, but significant, improvements in SNR, resulting in improved speech recognition in noise for adults (Ricketts & Hornsby 2003, 2006) and children (Hawkins & Yacullo 1984; Gravel et al. 1999; Ricketts et al. 2007; Ricketts & Picou 2013; Wolfe et al. 2022). However, the realized benefits of directional microphones depend on a number of factors, such as the spatial orientation between the speech, the background noise, and the listener (Ricketts et al. 2007; Ricketts & Picou 2013; Wolfe et al. 2017, 2022; Gustafson et al. 2021).

Another type of hearing aid feature that can affect listening in noise is single-microphone, digital noise reduction. Rather than using spatially sensitive microphones, digital noise reduction algorithms use the acoustic properties of a signal to classify it as primarily speech or primarily noise and then modify the gain accordingly, usually by reducing gain for signals classified as noise (Chung 2004; Hamacher et al. 2005; Bentler & Chiou 2006; Ricketts et al. 2019). Although the exact implementation of noise reduction algorithms varies considerably across, and even within, manufacturers (Hoetink et al. 2009; Scollie et al. 2016; Miller et al. 2017), the perceptual consequences of activating digital noise reduction are generally consistent within the literature. Specifically, digital noise reduction algorithms generally do not improve speech recognition performance, but have subjective benefits for listening comfort, as reported by adults (Chong & Jenstad 2018; Lakshmi et al. 2019).

### Digital Noise Reduction for School-Aged Children

Similarly, previous study with school-aged children in the laboratory demonstrates that digital noise reduction does not have significant effects on speech recognition performance (Stelmachowicz et al. 2010; McCreery et al. 2012), but children generally prefer to have it activated (Pittman & Hiipakka 2013; Wolfe et al. 2022) as long as the noise reduction algorithm is not too aggressive (Scollie et al. 2016). There is also some evidence to suggest digital noise reduction might reduce listening effort (Gustafson et al. 2014) and facilitate novel word learning (Pittman 2011a). However, the existing studies comparing outcomes with digital noise reduction activated compared with deactivated have several limitations. First, there are only a few studies that address this technology in school-aged children, and most of them were published more than a decade ago. Digital noise reduction technologies have advanced considerably in the interim (Lakshmi et al. 2019). For example, at least one commercial hearing aid manufacturer has an advanced form of digital noise reduction available that combines noise reduction with directivity (Herbert 2020).

Second, despite the established benefits of digital noise reduction being related primarily to subjective ratings of comfort or overall preference (Chung 2004; Hamacher et al. 2005; Bentler & Chiou 2006; Ricketts et al. 2019), many studies of digital noise reduction in children did not include a measure of preference or sound quality (Auriemmo et al. 2009; Pittman 2011a; Crukley & Scollie 2014). Among those that included children’s subjective preferences for digital noise reduction setting, only a single type of methodology was used in each study (Pittman & Hiipakka 2013; Scollie et al. 2016). For example, Pittman and Hiipakka (2013) played speech and noise and allowed participants to cycle through an orthogonal arrangement of programs that varied on directionality and noise reduction activations. A similar approach was used by Wolfe et al. (2022), who asked participants to listen to speech in noise and rank order their preferred noise cleaning programs, which also varied orthogonally by directivity and noise reduction activation. It is unclear if the conclusions from this type of task generalize to different measures of preference (e.g., double-blinded preference ratings).

Furthermore, in the extant literature, only three studies to date have included evaluations of digital noise reduction among children in the field, rather than in a laboratory. First, Crukley and Scollie (2014) evaluated directional microphones and digital noise reduction in combination in real classrooms, but used recorded test material in an otherwise empty room. Second, Wolfe et al. (2017) evaluated children’s preference for noise cleaning programs, which were a combination of directional microphones and digital noise reduction, during a field trial. Finally, Auriemmo et al. (2009) used parental and child questionnaires about hearing aid performance during a field trial. However, the questionnaire focused on performance, rather than preference for digital noise reduction. Therefore, it is not yet clear if laboratory findings regarding preference for digital noise reduction itself translate to real-world preferences for digital noise reduction.

### User Control

One factor that can affect the generalization of laboratory findings into real-world benefits is the degree to which digital noise reduction is active in the real world. Although most commercial implementations of digital noise reduction have automatic activations, the strength of the activation can be controlled in the programming software by a dispenser or in a smartphone application by the end user (for some hearing aid manufacturers). By manually controlling the digital noise reduction strength with a smartphone application, children might be able to optimize digital noise reduction in real-world environments. However, there has historically been some debate over the provision of manual access to noise-cleaning technologies for children.

On one hand, some authors advocate for children to have access to these noise cleaning features and to allow them to be in control of the algorithms (Pittman & Hiipakka 2013). Similarly, some audiologists provide manual control for noise cleaning algorithms for school-aged children, at least for cochlear implant users (Findlen et al. 2022). In addition, a recent study with school-aged children who wear hearing aids demonstrated that a smartphone application to control noise reduction and microphone directionality could be appropriate for children older than 10 yrs of age. Specifically, Gazibegovic et al. (2024) tested school-aged children in the laboratory and over a field trial, providing access to a smartphone application. They reported that children found the application easy to use and were able to self-adjust the noise cleaning settings in a way that optimized their speech in noise performance. Therefore, with the widespread adoption of smartphones (Picou 2020), even among school-aged children (Lenhart et al. 2015), it seems feasible to allow children to control their noise programs with a smartphone application.

On the other hand, there is some evidence that school-aged children do not manually access programs to optimize listening in noise. For example, using traditional push-button program changes on hearing aids, Ricketts et al. (2017) reported that no school-aged children in their study appropriately changed their manual program to activate (or deactivate) directional microphones during the school day, despite extensive training on switching before the field trial. This suggests that school-aged hearing aid users have limited capacity, or possibly motivation, to manually access noise cleaning programs, limiting their ability to optimize noise cleaning settings in the field.

Currently, most hearing aids being fitted to school-aged children are set by dispensers to default to an automatic setting, switching between noise cleaning settings automatically based on the environment (Nelson et al. 2024). It is unclear if school-aged children will be able to, or prefer to, override the automatic settings via manual control. Given the study by Gazibegovic et al. (2024) demonstrating success with a smartphone application, and the study of Ricketts et al. (2017) demonstrating no success with a manual button control, it seems likely that, if children are to be successful optimizing their noise cleaning features in real-world environments, it would be with a smartphone application. However, the extent to which school-aged children would use such control, or what their preferred settings would be in the real world, is largely unknown.

### Purpose

The purpose of this study was to evaluate the effects of an advanced digital noise reduction algorithm on measures in the laboratory (double-blind paired comparison testing, unblinded slider setting, sentence recognition performance) and during a field trial (unblinded slider setting). The current study involved laboratory procedures wherein participants provided subjective ratings of listening ease using a double-blinded, paired comparisons approach. They were also trained on the use of a smartphone application for manually controlling the advanced noise reduction algorithm. Their preferred setting set in the laboratory (unblinded) was compared with their preferred setting obtained in the blinded preference task, to evaluate the extent to which the different tasks provided converging evidence regarding listening preferences. Participants also completed behavioral sentence recognition in noise testing, also double-blinded, to evaluate the potential benefits of the advanced noise reduction algorithm on behavioral speech recognition performance. Based on previous literature (Chong & Jenstad 2018; Lakshmi et al. 2019), it was expected that digital noise reduction would benefit subjective ratings of preference, but not affect speech recognition performance.

Finally, participants wore the hearing aids at home during a field trial, with instructions to use the smartphone application during the trial and report on their experiences. The purpose of this field trial was to evaluate children’s preferences for advanced digital noise reduction to be active in the field, and also to evaluate their willingness to use a similar smartphone application to modify the strength of digital noise reduction in the future. It was expected that participants would prefer digital noise reduction to be active, even during the field trial. Given the study by Gazibegovic et al. (2024), it was also expected that they would be able to use the smartphone application with ease, prefer to be able to use it at home to control advanced digital noise reduction, and find it beneficial in noisy situations.

## MATERIALS AND METHODS

### Participants

**Fig. 1.**
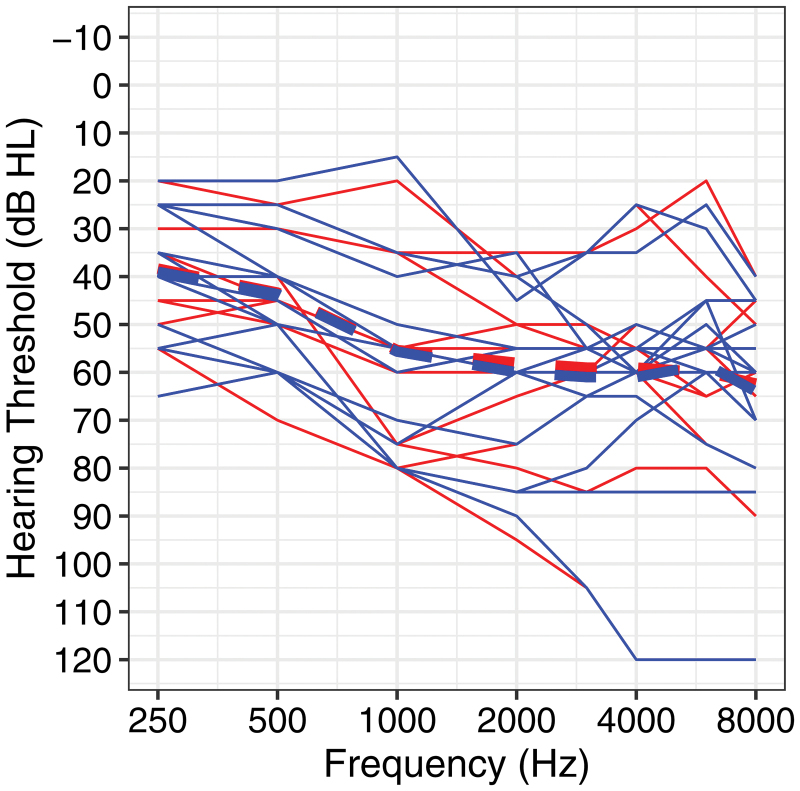
Individual pure-tone, air conduction audiometric thresholds for study participants. Red lines indicate right ear thresholds and blue lines indicate left ear thresholds. Thick dashed lines indicate the mean threshold at each frequency for each ear.

Eighteen children participated in the study (aged 10 to 17 years, mean *=* 14.3). However, three were withdrawn during or after study completion. Two participants (15- and 12-yr-old female) did not wear the study hearing aids during the field trial, and 1 participant did not complete all study visits (12-yr-old female). Therefore, descriptions and analyses are based on the 15 remaining participants (aged 10 to 17 years; mean = 14.6 years; 6 female and 9 male). All participants had longstanding bilateral, sensorineural hearing loss (see Fig. 1 for pure-tone, air conduction thresholds); the mean reported duration of hearing loss was 11.9 yrs (range = 2 to 17 yrs). Etiology was reported to be hereditary in 4 cases, congenital cytomegalovirus in 1 case, and was otherwise unknown (n = 10).

All participants were established hearing aid users who had worn hearing aids for more than 6 months before study enrollment (mean = 9.1 yrs, range = 2 to 16 yrs). By self-report, participants were using the hearing aids often, either almost always (n = 10) or most of the time (n = 3). Only 2 participants reported wearing hearing aids only “some of the time.” Most participants were bilateral hearing aid users; only one was a unilateral hearing aid user. Most of the participants (n = 12) reported they could control their hearing aid volume either on a smartphone or with a volume control button on the aids themselves. Three participants reported having no control over their hearing aid.

Potential participants were excluded from study participation if their hearing loss was asymmetrical (>30 dB difference between ears at 2 consecutive frequencies) or if their hearing loss was conductive or mixed (air-bone gaps larger than 20 dB at 2 consecutive audiometric frequencies). Potential participants were also excluded if they had a history of neurodevelopmental disorders, cognitive disorders, or speech-language disorders, as evidenced by parental report or documentation in the medical record. Participants were compensated for their time based on the number of laboratory visits and home trials they completed. This article describes a subset of procedures that was conducted during a larger protocol, which was preregistered (NCT05372094). Study procedures were conducted with approval by the Vanderbilt University Medical Center Institutional Review Board (#220482). The study was conducted between February and September 2023.

### Hearing Aid Fitting

For the purpose of the study, participants were fit with commercially available Phonak (Stafa, Switzerland) Lumity 90 hearing aids (receiver-in-the-canal) bilaterally. Within the programming software, the strength of the advanced digital noise reduction can vary from 0 to 20 (off to strongest). For laboratory testing, three manually accessible programs were created, which varied by advanced digital noise reduction setting (off, weak, and strong). Specifically, there were programs with the advanced noise reduction set to 0 (off), 8 (weak), and 20 (strong). These three settings were chosen to reflect the range of available options without testing every setting, which would be experimentally time-consuming. Supplemental Digital Content 1, https://links.lww.com/EANDH/B839, displays test box measures demonstrating that the algorithm has a minimal effect on the hearing aid response for speech in quiet or for signals originating in front, but does reduce hearing aid output for noise originating from the back over and above the microphone directivity.

Although all participants’ study hearing aids had programs with advanced digital noise reduction to be “off,” “weak,” and “strong,” the order was randomized across participants. That is, 1 participant would have program 1 set to be “off,” program 2 set to be “weak,” and program 3 set to be “strong.” A different participant would have programs 1, 2, and 3 set to be “strong,” “weak,” and “off,” respectively. The order of the programs was determined and programmed by a researcher who did not perform any laboratory testing to maintain double-blinding during laboratory testing; neither the participant nor the experimenter doing testing knew the strength of the advanced digital noise reduction being evaluated in each condition of the double-blinded tasks, where program order was randomized across participants (described in the following paragraph).

The manually accessible programs were defined as being for “Speech in Noise” by the hearing aid manufacturer. The programs had an adaptive directional microphone setting; nonlinear frequency compression was deactivated. The other default hearing aid settings were preserved in all hearing aid programs, including traditional noise reduction, wind noise, reverberation, and feedback reduction.

The hearing aid gain configurations for all programs were programmed individually for each participant and each ear to match the gain configurations of participants’ own hearing aids in response to a moderate-level signal. To do this, a probe microphone system (Audioscan Verifit 2; Dorchester, Canada) was used to measure the participants own hearing aids’ response to recorded speech (carrot passage at 65 dB SPL). Then, the research hearing aids were programmed to match the measured frequency response. The frequency response in the participants’ own hearing aids, the frequency response of the research hearing aids used in the study, and the prescribed hearing aid frequency response based on prescriptive targets (Desired Sensation Level 5.0; Scollie 2007) are displayed in Supplemental Digital Content 2, https://links.lww.com/EANDH/B840. The other details of the participants’ own hearing aids (e.g., feature activation) were not available to the researchers.

### Double-Blind, Paired Comparisons

To evaluate preference for the advanced digital noise reduction setting, participants completed paired comparison testing. This approach was similar to one used previously in adults (Wu et al. 2019) and has been shown to be appropriate for children as young as 5 yrs old (Eisenberg & Levitt 1991; Eisenberg & Dirks 1995; Ching et al. 2010). Participants listened to two sentence stimuli in one program (i.e., one advanced digital noise reduction setting), followed by the same two sentences in a second program (a different strength of advanced digital noise reduction) and rated their preference (first or second program). The two concatenated sentences were approximately 3 sec in duration. Following the presentation of two sentences in each program, the experimenter asked the participants to indicate their preferred program based on which program made it easier to listen to the sentences.

The sentence stimuli were always the same two sentences from the Connected Speech Test (Cox et al. 1987, 1988), specifically the first two sentences in the passage about “umbrellas.” The sentences were presented at 68 dBA from a loudspeaker (Tannoy System 6A; Coatbridge) positioned 1.5 m directly in front of the participant. There was also background noise present from four loudspeakers located 3.5 m from the participant at 45°, 135°, 225°, and 270°. The background noise was uncorrelated cafeteria noise with a summative, overall level of 68 dBA. The noise was speech-shaped, temporally modulated, and includes sounds of a busy restaurant and voices but without intelligible speech; it has been used previously in laboratory investigations of hearing aid settings in school-aged children (Ricketts & Picou 2013). The test environment was a moderately reverberant (T30 = 475 msec) laboratory (5.5 m × 6.5 m × 2.25 m) with the participant seated in the center of the room and the experimenter seated off to the side (approximately 90° and 2 m from the participant). The speech and noise levels were chosen based on pilot testing that confirmed with hearing aid data logging that the advanced digital noise reduction would be fully active, if set to “strong.”

Before the start of data collection, the participant practiced the paired comparison task three times using the same stimuli used for testing. Each possible comparison was made in both possible orders (e.g., “off” compared with “weak” and “weak” compared with “off”). After the practice comparisons, there were a total of 18 trials, with each individual setting presented 12 times, and each comparison (“off”-“weak,” “weak”-“strong,” “off”-“strong”) presented a total of 6 times. Paired comparison testing was double-blind in that neither the participant nor the experimenter knew which setting (off, weak, strong) was assigned to which manually accessible program in the smartphone application (1, 2, 3). The order of the programs was randomized across and within participants. The use of 3 advanced noise reduction strengths and 18 trials is supported by previous work evaluating noise reduction preferences in adults (Houben et al. 2023).

### Smartphone Application Setting

Participants were trained to manipulate the smartphone application and adjust the advanced digital noise reduction setting on a dedicated study smartphone (Apple iPhone 11). Training and testing occurred in the same reverberant laboratory environment as the paired comparison testing. First, they were briefly trained on the application itself and how to manipulate the slider that controls advanced digital noise reduction (called Speech Focus in the smartphone application). They were also instructed not to adjust the other sliders or settings in the application, including any other noise reduction slider or the volume controls. Then, they listened to a story presented from the front speaker 1.5 m from them. The speech was a fairy tale read by a female talker used previously in evaluations of hearing aid technologies (Picou et al. 2020a, b); it was presented at 70 dBA. The same uncorrelated cafeteria noise presented from 4 corner speakers (summated level of 68 dBA) from the paired comparisons task was used for the slider setting task. The participant listened to the story in the background noise for several minutes and then was instructed to adjust the advanced noise reduction (Speech Focus) slider in the smartphone application. They were instructed to adjust the setting to a level that they preferred and made listening easiest. After the participant indicated they set the slider to be in their preferred setting, the experimenter stopped the story and the background noise and saved the program as a new, unique custom program within the smartphone application. Then, the experimenter took a picture of the advanced digital noise reduction slider setting for later analysis.

### Double-Blind, Sentence Recognition in Noise

To evaluate the effects of digital noise reduction on sentence recognition performance in noise, the Hearing in Noise Test for Children (HINT-C; Gelnett et al. 1995) was used in the same reverberant laboratory space as the paired comparison and slider setting tasks. The HINT-C is an adaptive SNR test that targets the SNR threshold (in dB) where a participant can repeat 50% of the speech material. The sentences are spoken by a man at an adaptive level, based on participant responses. In the case of incorrect responses, the speech level is increased by 2 dB and is decreased by 2 dB for correct responses. To be considered correct, the participant must successfully repeat the entire sentence. The initial starting level was 72 dBA and the adaptive up/down procedure was completed with 16 sentences in a condition, following 4 practice sentences with a 4 dB step size. The level of the background noise was stable at 68 dBA. The background noise was the same uncorrelated, cafeteria noise described above for the paired comparisons testing and the smartphone slider setting. The dB SNR for 50% sentence recognition performance was established with each of 3 advanced digital noise reduction settings (off, weak, strong) using the 3 programs established by a second experimenter to maintain the double-blind nature of the testing.

### Field Trial and Questionnaires

After the laboratory tasks, the participants were sent home for a 1-week trial with the research hearing aids and the application installed on a smartphone. In most cases (n = 14), the application was installed on the participant’s personal device. Only 1 participant did not have access to a smartphone; they were provided one for the purpose of the study (Apple iPhone 11). Participants were reminded of how to use the smartphone application and were instructed to use the advanced digital noise reduction slider (Speech Focus) when it was noisy. They were asked to save a custom program in the application that saved their slider setting after they adjusted it in the noisy setting. They were told they could save as many custom programs as they wanted during the field trial, for example, if they were in multiple different noisy situations where they felt different slider settings were best. When they returned from the field trial, they showed their saved custom programs to the experimenter, who took a picture of the saved slider settings created during the field trial. Then, they repeated the in-laboratory adjustments of the advanced digital noise reduction slider within the smartphone application in the reverberant laboratory. The experimenter then took a picture of their second preferred slider setting from the repeated laboratory task.

Following the field trial with the research hearing aids and smartphone application, participants completed a bespoke questionnaire related to their experiences with the smartphone application. The questions, displayed in Supplemental Digital Content 3, https://links.lww.com/EANDH/B841, asked about whether the participants used the smartphone application, if they liked using it, and if they would use it in the future. Questions were also asked about self-reported confidence and anxiety; responses to these questions are not included in this paper. All response options were verbal descriptions ranging from “not at all” to “a lot,” but with words specific to the question (e.g., “I really didn’t like it” to “I liked it a lot”). After each question, participants could enter free-response text to explain their answer, if desired. Participants completed the questionnaire in RedCap (Harris et al. 2009, 2019) in a quiet, clinic-like laboratory space with the experimenter available to answer questions.

### Procedures

Study procedures occurred over the course of 3 laboratory visits with the field trial occurring between study visits 1 and 2. During the first laboratory visit, guardians provided informed consent and participants provided assent to participate. Then, pure-tone air conduction thresholds were measured, and the research hearing aids were fit. Following the hearing aid fitting, participants completed the laboratory tests of double-blind paired comparison testing and smartphone slider setting. Before the conclusion of the first visit and the beginning of the field trial, the manually accessible programs were removed from the hearing aids and they were set to be fully automatic, switching between microphone and noise reduction settings based on the listeners’ environment. The experimenter ensured the smartphone and the hearing aids were paired and the participant could control the advanced digital noise reduction setting with the smartphone application. Participants returned approximately 1 week later.

During the second visit, participants completed the post-field trial questionnaire and showed the experimenter their final preferred digital noise reduction slider settings from the field trial. They then repeated the smartphone setting procedures to set their customized advanced digital noise reduction preference in the laboratory, as in the first study visit. The participants then completed laboratory tests of listening effort and wireless streaming, in addition to a second field trial, none of which are described in the current paper. Finally, participants returned for a third and final visit. During this visit, the manual programs were restored and participants completed double-blind, sentence recognition in noise testing in the laboratory. At the conclusion of the visit, they returned the hearing aids and were not given the opportunity to purchase them.

### Data Analysis

All data analyses and figure generations were conducted in R language for statistical computing (v. 4.3.0; R Core Team 2023). Data for each task were analyzed separately (double-blind paired comparison preferences, smartphone application setting, sentence recognition in noise performance, post-field trial questionnaire responses). The analytic approach and specific statistical packages used in the analyses for each task are discussed in detail later. For all analyses, significance was defined as *p* < 0.05 and adjustments were made to control for family-wise error rate (Benjamini & Hochberg 1995). In addition to the analyses presented earlier, exploratory analyses were conducted to investigate how preferences might be related to individual factors (e.g., age, pure-tone average, duration of hearing aid experience, experience with hearing aid user controls). Analyses did not reveal any statistically significant relationships between individual factors and preferences; the results are thus not reported in the article.

## RESULTS

### Double-Blind, Paired Comparisons

**Fig. 2.**
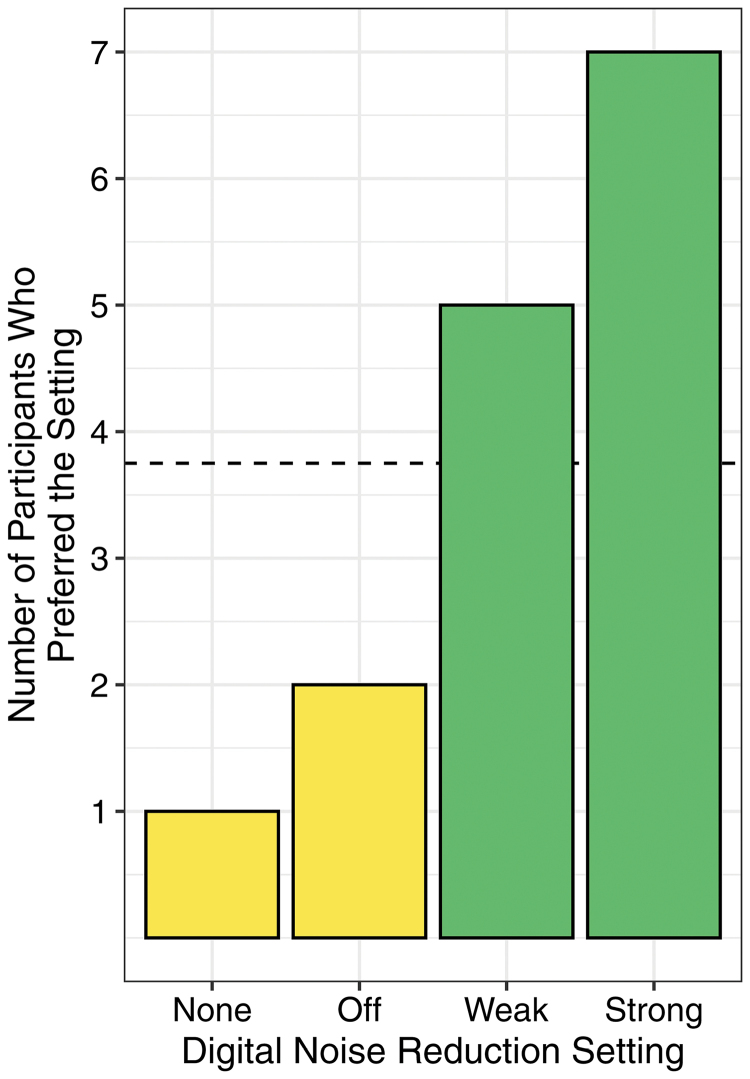
Number of participants who preferred each digital noise reduction setting during the double-blinded, paired comparisons testing. The dashed line indicates the number of participants who would prefer the setting, if each setting was equally preferred by all participants. Yellow bars indicate the settings that were combined for statistical analysis to indicate “digital noise reduction not preferred” (no preference or off) and green bars indicate settings (weak or strong) that were combined for statistical analysis to indicate “advanced digital noise reduction preferred.”

The setting that each participant chose the highest number of times (chosen as the preferred setting from the pair in the paired comparison) was assigned to be their ultimate preferred program. For example, their preferred program would be determined to be “weak” if they selected that program in 75% of the trials where it was presented against any other setting of advanced digital noise reduction. Scores of 50% represent no preference because it indicates a participant chose the setting only half of the time it was presented compared with any other setting. In the paired comparison approach, that means they would have chosen the other option just as frequently. Only one participant chose all settings in 50% of trials; they were assigned as having “no preference.” The number of participants who preferred each advanced digital noise reduction setting (off, weak, strong) or had no preference during the paired comparisons testing is displayed in Figure 2. This figure reveals that most participants (n = 12; 80%) preferred advanced digital noise reduction to be active, either at the weak setting (n = 5) or at the strong setting (n = 7). Conversely, only 1 participant had no preference, and 2 participants preferred digital noise reduction to be off.

A χ^2^ goodness of fit test was used to evaluate whether all preferences identified during the paired comparisons testing were equally common; the test was conducted using the **chi.square** function in base R and simulated *p* values. Due to the relatively small sample size, the participant with no preference and those who preferred noise reduction to be “off” were grouped together. Similarly, participants whose preferred program was active (either weak or strong) were grouped together. The null hypothesis was that digital noise reduction preferred (weak/strong), and digital noise reduction not preferred (off/no preference) would result in equal probability (50%) each. The statistical test results rejected the null hypothesis; there were more participants who preferred the advanced noise reduction setting to be active than there were participants who had no preference or preferred it to be off (Χ^2^ = 5.4, *p* = 0.039).

### Smartphone Application Setting

**Fig. 3.**
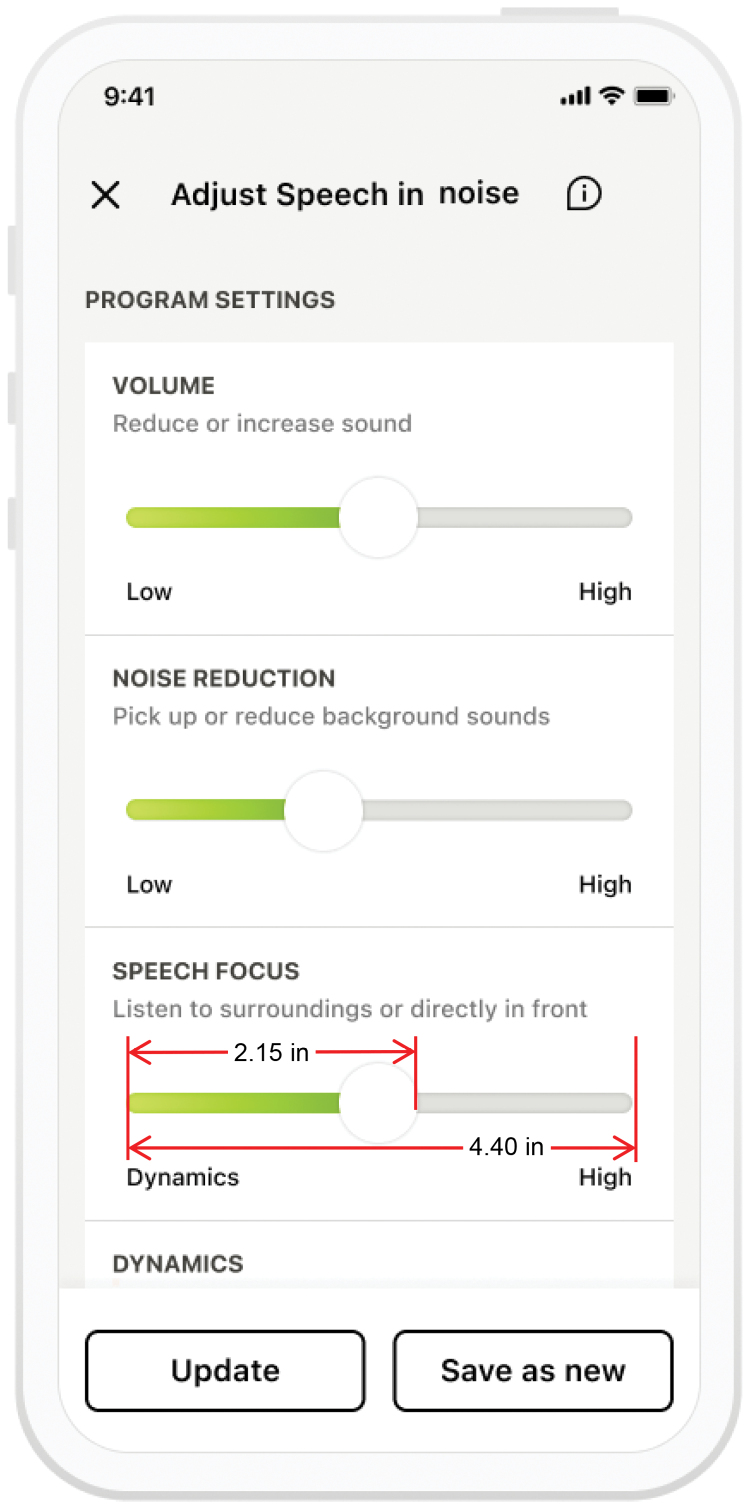
Example screen capture of the smartphone application and the advanced digital noise reduction slider. The figure also displays the measurement technique for calculating the preferred slider setting (e.g., measuring the location of the indicator compared with the full width of the slider). In this example, the preferred slider setting was calculated to be 49% (100 × 2.15/4.40). This position would indicate that advanced noise reduction is “off” in the hearing aids.

To evaluate the preferred setting of the advanced digital noise reduction slider (Speech Focus) setting in the smartphone application, the pictures of the sliders were converted to portable document format (Adobe Acrobat). Using the measuring tool in Adobe Acrobat, the relative position of the slider setting was compared with the overall width of the slider. The percent of the slider activated at the preferred setting was defined as the width of the slider at the preferred setting divided by the full width of the slider (and multiplied by 100). For all participants, the rightmost slider edge was used for this measurement. As a result, 100% slider setting indicates the slider was in the far-right position and that advanced digital noise reduction was fully active. However, a slider score of 57% indicates the indicator was in the middle of the slider, due to the use of the rightmost edge of the indicator and due to the large size of the slider indicator. An example smartphone application screen capture, indicator, slider, and measurement are displayed in Figure 3. A slider position of 100% indicates advanced digital noise reduction setting is “strong.” Slider positions less than 57% indicate that the advanced digital noise reduction is deactivated. Further movements to the left (less than 57%) reduce the directionality of the hearing aid microphones, with a far-left position (0%) setting the hearing aids to be fully omnidirectional.

Recall that participants indicated their preferred advanced digital noise reduction setting with the slider three times (visit 1 in the laboratory, saved program in the field, visit 2 in the laboratory). The mean preferred slider position for visit 1 in the laboratory was 71.3% (SD = 24.2), after the field trial, the mean preferred slider position was 60.1% (SD = 27.2), and for visit 2 in the laboratory, the mean preferred slider position was 77.5% (SD = 16.6). To evaluate whether the preferred slider setting was different than “off” (as indicated by slider setting of 57%), the **emmeans** function of the *emmeans* package (Lenth 2019) was used to test the settings at each visit against the null hypothesis of 57%. Results indicated that the slider settings were significantly greater than 57% for both of the laboratory visits (*p* < 0.01), but not in field situations (*p* = 0.265). These results indicate that, on average, participants preferred advanced digital noise reduction to be active when measured in the laboratory visits but did not prefer the slider to have advanced digital noise reduction active in the noisy situations in the field. Note that 4 participants could not save a preferred setting in the field (due to technical difficulties with the application [n = 2] and due to not following instructions [n = 2]). In the paired comparison testing, all 4 of these participants preferred to have noise reduction active (3 preferred “strong” and 1 preferred “weak”).

**Fig. 4.**
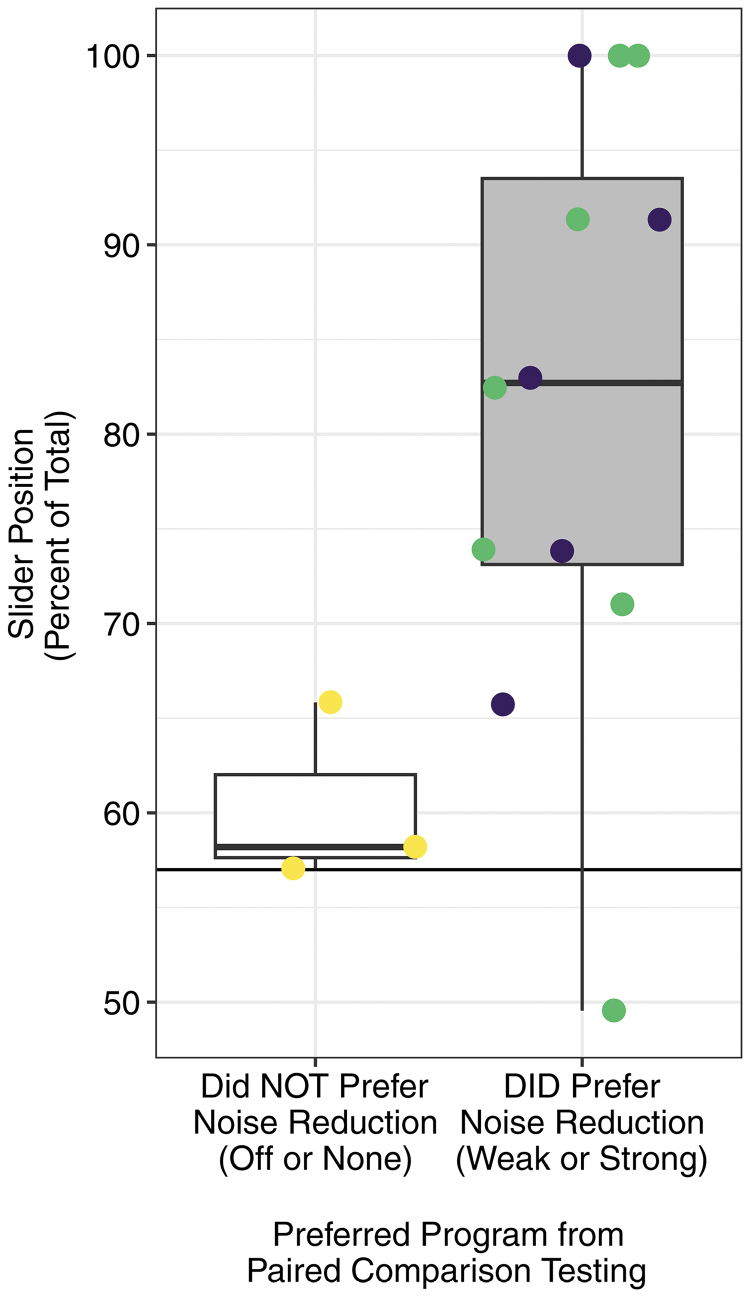
Preferred slider setting during second laboratory test session for participants whose preferred setting was determined during double-blinded, paired comparison testing was digital noise reduction off (or no preference) and for those whose preferred setting was digital noise reduction on (either weak or strong, purple and green dots, respectively). Those whose preferred digital noise reduction setting was on also set their application slider to have digital noise reduction to be stronger than did those who did not prefer noise reduction setting to be active. Horizontal line indicates a slider setting in the middle, which would indicate digital noise reduction is not active.

To evaluate the consistency of the two types of preference-related laboratory tasks (the application slider setting [not blinded] and the paired comparison testing [double-blinded]), the final preferred slider setting in the laboratory was compared between participants who did not prefer advanced digital noise reduction to be active (off/no preference) to the setting of participants who did prefer advanced digital noise reduction to be active (weak/strong), as measured during double-blind, paired comparison testing (Fig. 4). Linear modeling with preference (off/none or weak/strong) as a between-group factor revealed a significant difference in calculated slider settings between groups [*F*(1,13) = 5.21; *p* < 0.05]. Comparisons using **emmeans** revealed that participants with a preference for advanced digital noise reduction during paired comparison testing also set the slider higher (estimated marginal mean = 81.8%) than did participants without a preference for digital noise reduction to be active (estimated marginal mean = 60.4%). These data support consistency in results between the two types of preference measures in the noisy, reverberant laboratory setting (double-blinded paired comparisons and unblinded slider setting).

### Sentence Recognition in Noise

Table 1 displays the sentence recognition in noise scores (dB SNR that results in 50% sentence recognition performance with each level of advanced digital noise reduction). Sentence recognition in noise scores were analyzed using linear mixed-effects models with advanced digital noise reduction setting (off, weak, strong) as a fixed factor and participant as a random intercept. The model was constructed using the **lmer** function of the *lme4* package (Bates et al. 2015). Analysis of the model using the *anova_stats* function of the **sjstats** package (Lüdecke & Lüdecke 2019) revealed that the advanced digital noise reduction setting did not significantly affect sentence recognition scores [*F*(3,42) = 1.31; *p* = 0.283]. Differences between conditions were small (<0.02 dB) and when accounting for random effects, the size of the effect related to the advanced digital noise reduction algorithm on speech recognition in noise performance was small (*η*_p_^2^ = 0.09).

**TABLE 1.** Sentence recognition in noise scores (dB SNR required for 50% sentence recognition) with each advanced digital noise reduction setting

Digital Noise Reduction Setting	Mean	SD	Range
Off	1.01	0.05	0.92–1.13
Mild	1.02	0.05	0.95–1.14
Strong	1.02	0.06	0.94–1.17

Differences between advanced digital noise reduction settings were not statistically significant.

### Post-Field Trial Questionnaire

**Fig. 5.**
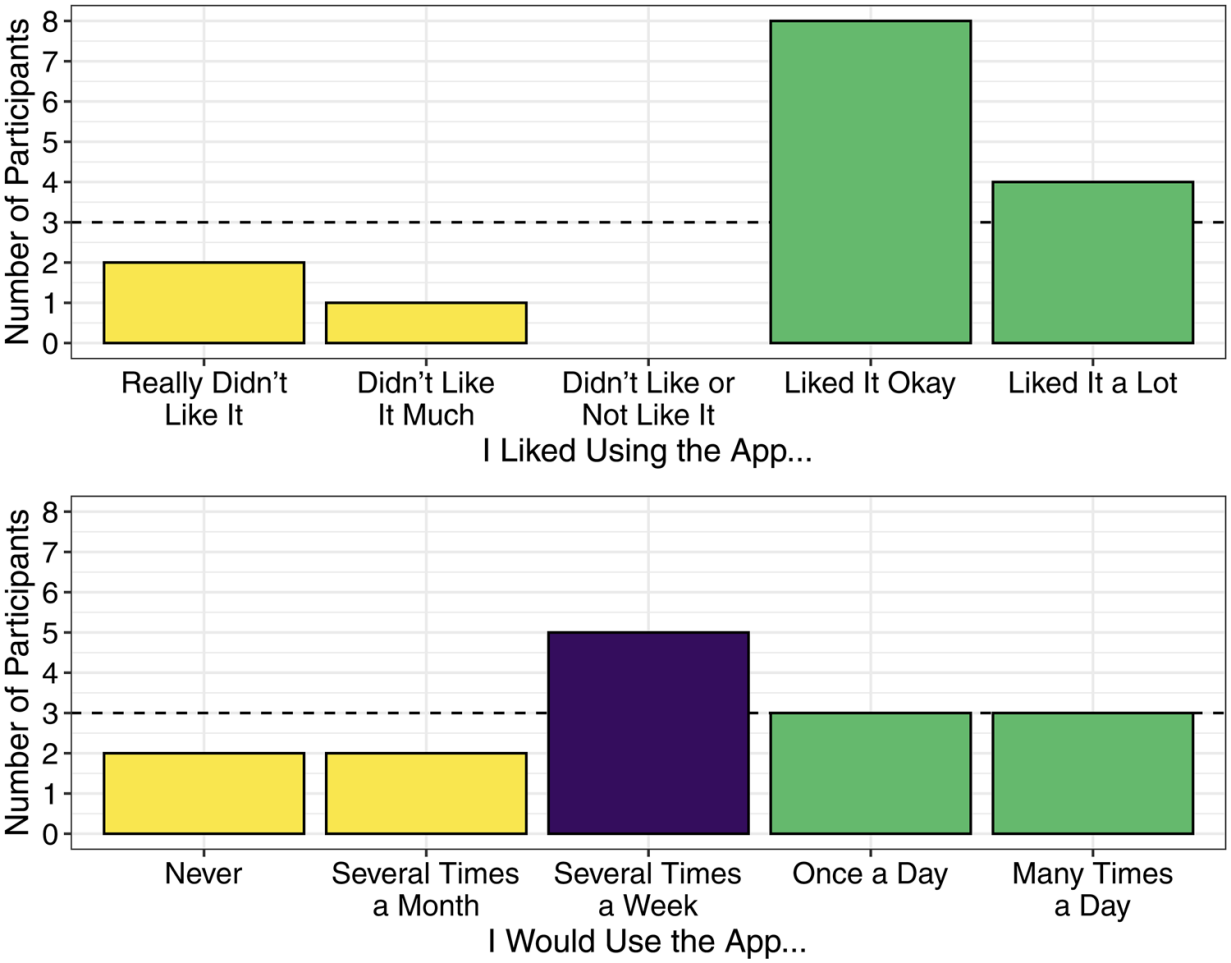
Number of participants who responded with each response option related to liking the use of the slider (top panel) and plan to use it in the future (bottom panel). Yellow bars indicate responses that are generally unfavorable toward the slider, purple bars reflect responses that are neutral, and green bars reflect responses that are generally favorable toward the slider. Note that the sample size was 15 for both panels.

Responses to the question about smartphone application use (did you use the slider in the smartphone application?) indicated that all participants used it at least once (n = 15). Although not explicitly asked about the listening environment, inspection of the free response explanations revealed that most participants reported using the application to adjust the noise reduction algorithm in a noisy place (e.g., a restaurant, n = 9). One participant reported they “did not go out much” and reported not trying it in a noisy place. Five participants did not comment on their auditory environment during the field trial. Participants also varied in the frequency of the application use. Four participants reported using it “once or twice,” another four reported using it “several times” in a week, three reported using it “once a day,” and four reported using it “many times a day.” Because all participants reported using the application at least once, all data were evaluated for the questions about whether they liked using the slider in the smartphone application (Fig. 5, top panel) and if they would use the application in the future (Fig. 5, bottom panel).

For each of these questions, Chi-squared goodness-of-fit testing was completed using the **chisq.test** function. The null hypothesis was defined as equal probabilities of unfavorable ratings (e.g., “really didn’t like it” and “didn’t like it much”), neutral ratings (e.g., “didn’t like it or not like it”), and favorable ratings (e.g., “liked it okay” and “liked it a lot”). Analysis of the question related to liking the application revealed that the responses were not equally likely (Χ^2^ = 10.5; *p* = 0.005); more participants reported liking the application than reported not liking it. However, analysis of intent to use the application in the future did not reject the null hypothesis (Χ^2^ = 2.0; *p* = 0.368), suggesting that all three categories of response options (unfavorable, neutral, favorable) were equally likely. These data demonstrate that, although participants report liking the slider in the smartphone application, they were just as likely to use as not to use the application in the future.

## DISCUSSION

The purpose of this study was to evaluate the effects of an advanced digital noise reduction algorithm on measures in the laboratory (double-blind paired comparison testing, unblinded slider setting, sentence recognition performance) and during a field trial (unblinded slider setting). A secondary purpose was to evaluate participants’ preference for using a smartphone application for controlling digital noise reduction in the field and their reported willingness to use the application in the future.

### Digital Noise Reduction in the Laboratory

The current study demonstrates converging lines of evidence from different tasks that school-aged children preferred advanced digital noise reduction to be active in a noisy, reverberant laboratory environment. First, most participants (80%) chose digital noise reduction to be active during the double-blind, paired comparison testing (Fig. 2). Second, in the laboratory, participants chose digital noise reduction strength that was active using the advanced digital noise reduction slider in the smartphone application (on average, 74% of the slider, where 57% indicates advanced digital noise reduction is deactivated and 100% indicates advanced digital noise reduction is on “strong”). The finding that children in the current study indicate similar preferences in the blinded and unblinded laboratory tasks is generally consistent with the assertions by some that children can give reliable preference ratings for noise management strategies (Pittman & Hiipakka 2013) and other hearing aid features (Parsa et al. 2013).

It is important to note that there was agreement between these two outcomes, not only in overall effects, but also at an individual level. Most participants who preferred advanced digital noise reduction to be active (weak or strong) in the paired comparison testing also set the advanced digital noise reduction slider in the application to have digital noise reduction activated (Fig. 4). Conversely, the few participants who did not prefer advanced digital noise reduction to be active (off or no preference), also chose slider settings with digital noise reduction deactivated (position less than 57% on the smartphone application). This internal consistency is remarkable due to the nature of the two tasks. Although the slider setting task might be prone to biases associated with studying novel hearing aid features, such as a placebo effect (Dawes et al. 2013) or a labeling effect (Bentler et al. 2003), the double-blind, paired comparison testing would not be as sensitive to these biases. The consistency between the measures lends credence to both tasks and suggests that the adolescents in the study were consistent in their preferences and minimally influenced by the procedures themselves to choose the “best” setting.

As expected, the preference for digital noise reduction to be activated was not associated with improvements in sentence recognition performance. Instead, sentence recognition scores, using an adaptive HINT-C test stimuli, were unrelated to the strength of digital noise reduction. Differences between conditions were very small (~0.01 dB, on average; Table 1). These data indicate that the perceptual benefits for advanced digital noise reduction are specific to subjective effects of ease of listening and overall preference. This is consistent with extant literature demonstrating subjective benefits with traditional digital noise reduction without large changes in speech recognition performance in adults (Lakshmi et al. 2019) and children with hearing loss (Chong & Jenstad 2018). It is also consistent with the function of the advanced digital noise reduction; as described in Supplemental Digital Content 1, https://links.lww.com/EANDH/B839, the feature reduces hearing aid output only for signals arriving from the back and does not affect speech in quiet or signals arriving from the front.

### Digital Noise Reduction at Home

Unlike the laboratory measures of advanced digital noise reduction preferences, the slider settings participants reported preferring while listening in specific situations in the field did not indicate that participants preferred to activate the advanced digital noise reduction using the smartphone application. That is, the custom program that participants saved during a difficult listening experience and brought to the laboratory after the field trial, on average, was set to 60% of full-on noise reduction activation, which was not significantly different from noise reduction deactivated (57% of full slider length). The reasons for the disconnect between the robust preferences most participants had in the laboratory and the nonsignificant benefit of advanced digital noise reduction during the field trial are unclear and are likely multifaceted. For example, recall that technological issues prohibited the creation of a final, preferred digital noise reduction setting for 4 of the 15 participants. Among these 4 participants, 3 preferred “strong” and 1 preferred “weak” digital noise reduction settings in the laboratory. It is possible that the exclusion of their preferred slider settings has affected the reconciliation of the laboratory and field results.

It is possible that the listening environments where participants used the smartphone application in the field were more favorable (less noisy) than the ones they encountered in the laboratory. Classrooms are not reflective of most laboratory listening situations (Mealings et al. 2024). Furthermore, participants were encouraged to wear the study hearing aids full-time and to use the slider in the application in a noisy, difficult listening situation. However, it is clear that not all participants followed this instruction. Although most participants (n = 9) volunteered the information that they used the slider in a noisy listening situation, the remaining 6 participants either did not use the slider in noise or did not comment on how they used it. Given that most participants reported that they would use the smartphone application infrequently, it is possible that the settings reflect that they did not change the slider from default, rather than a strong preference for the digital noise reduction to be deactivated in the field.

It is also possible that the children did not use the slider during the field trial in a way that was meaningful or allowed for the optimal setting. It has been established that children are unlikely to modify their hearing aid settings via push buttons (Ricketts et al. 2017). In addition, although children reported using their smartphone applications to adjust the slider in the current study, they did not do so very often. It is also possible that most children in the study did not have experience changing their hearing aid settings with a smartphone and thus were not in the habit of making adjustments with a smartphone. Only 2 study participants reported having access to a smartphone application to control hearing aids before study enrollment.

One of the important findings from the current study is that children reported generally liking the smartphone application but reported they would not use it very often (Fig. 5), at least for the specific use case of changing the strength of the advanced digital noise reduction. These results are somewhat more optimistic than previous results demonstrating children never interacted with a manual control of hearing aid settings during the day (Ricketts et al. 2017), but still suggest resistance to manually accessing hearing aid controls, likely due to multifaceted reasons described earlier.

### Study Limitations and Future Directions

Although the study demonstrates robust benefits in the laboratory of advanced digital noise reduction and potential benefits for the smartphone application in the field, there are some important limitations to consider. First, there was no experimental control over the field trial for advanced digital noise reduction. Participants were simply encouraged to use the hearing aids and the smartphone in noisy situations, but it is possible they were not in noisy situations or did not use the smartphone application appropriately. Based on their self-report, all participants indicated they did try the smartphone application and controlled the advanced noise reduction during the field trial. In addition, the majority reported using it specifically in noisy situations. However, the nature and location of the noises were not documented. Future studies would benefit from more directed instructions or listening situations to ensure that participants use the noise reduction algorithm in a noisy place. Data logging or dosimetry would be a valuable complement to such instructions to facilitate the interpretation of field trial data.

Second, to provide similar listening experiences for participants with the study hearing aids and their own hearing aids, the research aids were programmed to match the participants’ own frequency responses, not a validated gain prescription. As displayed in Supplemental Digital Content 2, https://links.lww.com/EANDH/B840, participants’ own hearing aids were generally under-fit relative to prescriptive gain targets. It is possible that the effects of digital noise reduction would be different for hearing aids that were matched to prescriptive targets (e.g., DSL; Scollie 2007). By using a validated prescriptive fitting method that optimizes audibility, advanced digital noise reduction might be more beneficial.

Finally, the current study did not include open-ended, subjective feedback from participants about advanced digital noise reduction in the laboratory. It is not clear why 13% of participants (n = 2 of 15) preferred to have digital noise control deactivated. In addition, the free response feedback in the field trial was not required. Not all participants provided responses explaining their answers. Qualitative data about the advanced digital noise reduction algorithm and its control with the smartphone application could help elucidate mechanisms for improved subjective quality with the algorithm active and could also help predict which patients would prefer not to have digital noise reduction activated. In future study, qualitative analysis of subjective reports of advanced digital noise reduction would provide a complement to the quantitative data in the current study.

## CONCLUSIONS

The results of the current study demonstrate robust subjective benefits of advanced digital noise reduction in the laboratory for children, with minimal effects on speech intelligibility. In addition, the findings with the smartphone application demonstrate that, although school-aged children were unlikely to make changes many times a day, many reported willingness to use the smartphone application in the future at least occasionally. Participants did not generally prefer to have digital noise reduction activated during specific listening situations in the field trial, although this finding could have been affected by the auditory lives of the participants during the field trial. Future study is warranted to reconcile the laboratory and field trial findings in this study. In the interim, given the preferences for the algorithm and the limited speech recognition consequences, activating advanced digital noise reduction by default, perhaps even on the weak strength, would be a reasonable clinical approach for school-aged children. By providing a smartphone application, the minority of patients who do not prefer the algorithm to be active could control the advanced digital noise reduction.

## ACKNOWLEDGMENTS

The authors thank Rachel Kruse for assistance in data collection.

Andrea Dunn is currently at the Oberkotter Foundation.

Sonova provided research support for the project.

## Supplementary Material

## References

[R1] American National Standards Institute. (2010). Acoustical Performance Criteria, Design Requirements and Guidelines for Schools, Part 1: Permanent Schools. 12.60-2010/Part 1.

[R2] Auriemmo J. Kuk F. Lau C. Dornan B. K. Sweeton S. Marshall S. Pikora M. Quick D. Thiele D. Stenger P. (2009). Efficacy of an adaptive directional microphone and a noise reduction system for school-aged children. J Educ Audiol, 15, 15–27.

[R3] Bates D. Maechler M. Bolker B. Walker S. (2015). Fitting linear mixed-effects models using {lme4}. J Stat Softw, 67, 1–48.

[R4] Benjamini Y., & Hochberg Y. (1995). Controlling the false discovery rate: A practical and powerful approach to multiple testing. J R Stat Soc Series B Stat Methodol, 57, 289–300.

[R5] Bentler R., & Chiou L. -K. (2006). Digital noise reduction: An overview. Trends Amplif, 10, 67–82. 16959731 10.1177/1084713806289514 PMC4111515

[R6] Bentler R. A. Niebuhr D. P. Johnson T. A. Flamme G. A. (2003). Impact of digital labeling on outcome measures. Ear Hear, 24, 215–224. 12799543 10.1097/01.AUD.0000069228.46916.92

[R7] Bess F. H. Sinclair J. S. Riggs D. E. (1984). Group amplification in schools for the hearing impaired. Ear Hear, 5, 138–144. 6734964 10.1097/00003446-198405000-00004

[R8] Browning J. M. Buss E. Flaherty M. Vallier T. Leibold L. J. (2019). Effects of adaptive hearing aid directionality and noise reduction on masked speech recognition for children who are hard of hearing. Am J Audiol, 28, 101–113. 30938559 10.1044/2018_AJA-18-0045

[R9] Ching T. Y. Scollie S. D. Dillon H. Seewald R. Britton L. Steinberg J. Gilliver M. King K. A. (2010). Evaluation of the NAL-NL1 and the DSL v. 4.1 prescriptions for children: Paired-comparison intelligibility judgments and functional performance ratings. Int J Audiol, 49, S35–S48. 20109087 10.3109/14992020903095791

[R10] Chong F. Y., & Jenstad L. M. (2018). A critical review of hearing-aid single-microphone noise-reduction studies in adults and children. Disabil Rehabil Assist Technol, 13, 600–608. 29072542 10.1080/17483107.2017.1392619

[R11] Chung K. (2004). Challenges and recent developments in hearing aids. Part I. Speech understanding in noise, microphone technologies and noise reduction algorithms. Trends Amplif, 8, 83–124. 15678225 10.1177/108471380400800302 PMC4111442

[R12] Cox R. M. Alexander G. C. Gilmore C. (1987). Development of the connected speech test (CST). Ear Hear, 8, 119S–126S. 3678650 10.1097/00003446-198710001-00010

[R13] Cox R. M. Alexander G. C. Gilmore C. Pusakulich K. M. (1988). Use of the Connected Speech Test (CST) with hearing-impaired listeners. Ear Hear, 9, 198–207. 3169400 10.1097/00003446-198808000-00005

[R14] Crandell C. C., & Smaldino J. J. (2000). Classroom acoustics for children with normal hearing and with hearing impairment. Lang Speech Hear Serv Sch, 31, 362–370. 27764475 10.1044/0161-1461.3104.362

[R15] Crukley J., & Scollie S. D. (2014). The effects of digital signal processing features on children’s speech recognition and loudness perception. Am J Audiol, 23, 99–115. 24018572 10.1044/1059-0889(2013/13-0024)

[R16] Crukley J. Scollie S. Parsa V. (2011). An exploration of non-quiet listening at school. J. educ. audiol, 17, 23–35.

[R17] Dawes P. Hopkins R. Munro K. J. (2013). Placebo effects in hearing-aid trials are reliable. Int J Audiol, 52, 472–477. 23594421 10.3109/14992027.2013.783718

[R18] Eisenberg L. S., & Dirks D. D. (1995). Reliability and sensitivity of paired comparisons and category rating in children. J Speech Hear Res, 38, 1157–1167. 8558884 10.1044/jshr.3805.1157

[R19] Eisenberg L. S., & Levitt H. (1991). Paired comparison judgments for hearing aid selection in children. Ear Hear, 12, 417–430. 1797609 10.1097/00003446-199112000-00006

[R20] Findlen U. M. Benedict J. Agrawal S. (2022). Clinical practice patterns of fitting advanced device features in children with cochlear implants. J Speech Lang Hear Res, 65, 797–815. 35015974 10.1044/2021_JSLHR-21-00168

[R21] Gazibegovic D. Bohnert A. Laessig A. K. (2024). Hearing Aid apps: Are they safe, practical and beneficial for children and teens in challenging situations? Eur Arch Otorhinolaryngol, 281, 6021–6029. 39083059 10.1007/s00405-024-08851-2 PMC11512926

[R22] Gelnett D. Sumida A. Nilsson M. Soli S. (1995). Development of the Hearing In Noise Test for Children (HINT-C). Paper presented at the American Academy of Audiology, Dallas, TX.

[R23] Gravel J. S. Fausel N. Liskow C. Chobot J. (1999). Children’s speech recognition in noise using omni-directional and dual-microphone hearing aid technology. Ear Hear, 20, 1–11. 10037061 10.1097/00003446-199902000-00001

[R24] Gustafson S. McCreery R. Hoover B. Kopun J. G. Stelmachowicz P. (2014). Listening effort and perceived clarity for normal-hearing children with the use of digital noise reduction. Ear Hear, 35, 183–194. 24473240 10.1097/01.aud.0000440715.85844.b8 PMC4060443

[R25] Gustafson S. J. Ricketts T. A. Picou E. M. (2021). Individual differences offer insight into clinical recommendations for directional and remote microphone technology use in children. J Speech Lang Hear Res, 64, 635–650. 33465321 10.1044/2020_JSLHR-20-00281

[R26] Hamacher V. Chalupper J. Eggers J. Fischer E. Kornagel U. Puder H. Rass U. (2005). Signal processing in high-end hearing aids: state of the art, challenges, and future trends. EURASIP J Appl Signal Process, 2005, 2915–2929.

[R27] Harris P. A. Taylor R. Thielke R. Payne J. Gonzalez N. Conde J. G. (2009). Research electronic data capture (REDCap)—A metadata-driven methodology and workflow process for providing translational research informatics support. J Biomed Inform, 42, 377–381. 18929686 10.1016/j.jbi.2008.08.010 PMC2700030

[R28] Harris P. A. Minor B. Elliott V. Fernandez M. O’Neal L. McLeod L. Delacqua G Delacqua F Kirby J Duda SN. (2019). The REDCap consortium: Building an international community of software partners. J Biomed Inform, 95, 103208. 31078660 10.1016/j.jbi.2019.103208 PMC7254481

[R29] Hawkins D. B., & Yacullo W. S. (1984). Signal-to-noise ratio advantage of binaural hearing aids and directional microphones under different levels of reverberation. J Speech Hear Disord, 49, 278–286. 6748623 10.1044/jshd.4903.278

[R30] Herbert A. (2020). Are you a noise hater? Retrieved January 10, 2025. https://audiologyblog.phonakpro.com/are-you-a-noise-hater/

[R31] Hoetink A. E. Körössy L. Dreschler W. A. (2009). Classification of steady state gain reduction produced by amplitude modulation based noise reduction in digital hearing aids. Int J Audiol, 48, 444–455. 19925331 10.1080/14992020902725539

[R32] Houben R. Reinten I. Dreschler W. A. Mathijssen R. Dijkstra T. M. (2023). Preferred strength of noise reduction for normally hearing and hearing-impaired listeners. Trends Hear, 27. doi:10.1177/23312165231211437. 10.1177/23312165231211437 PMC10666719 37990543

[R33] Lakshmi M. S. K. Rout A. O’Donoghue C. R. (2019). A systematic review and meta-analysis of digital noise reduction hearing aids in adults. Disabil Rehabil Assist Technol, 16, 120–129. 31502900 10.1080/17483107.2019.1642394

[R34] Lenhart A. Duggan M. Perrin A. Stepler R. Rainie H., & Parker K. (2015). Teens, social media & technology overview 2015.

[R35] Lenth R. (2019). emmeans: Estimated Marginal Means, aka Least-Squares Means. R package version 1.4. https://CRAN.R-project.org/package=emmeans.

[R36] Lüdecke D., & Lüdecke M. D. (2019). Package ‘sjstats’. Statistical functions for Regression Models, Version 0.17, 3.

[R37] McCreery R. W. Venediktov R. A. Coleman J. J. Leech H. M. (2012). An evidence-based systematic review of directional microphones and digital noise reduction hearing aids in school-age children with hearing loss. Am J Audiol, 21, 295–312. 22858614 10.1044/1059-0889(2012/12-0014) PMC3723379

[R38] Mealings K. Miles K. Buchholz J. M. (2024). A methodological review of stimuli used for classroom speech-in-noise tests. J Speech Lang Hear Res, 67, 4850–4866. 39560502 10.1044/2024_JSLHR-24-00261

[R39] Miller C. W. Bentler R. A. Wu Y. -H. Lewis J. Tremblay K. (2017). Output signal-to-noise ratio and speech perception in noise: Effects of algorithm. Int J Audiol, 56, 568–579. 28355951 10.1080/14992027.2017.1305128 PMC6076442

[R40] Nelson J. Pelosi A. Bulut K. Jagoda L. (2024). Using large-scale data analytics to understand pediatric hearing aid prescription and use. Hear Rev, 31, 16–19.

[R41] Neuman A. C. Wróblewski M. Hajicek J. Rubinstein A. (2010). Combined effects of noise and reverberation on speech recognition performance of normal-hearing children and adults. Ear Hear, 31, 336–344. 20215967 10.1097/AUD.0b013e3181d3d514

[R42] Parsa V. Scollie S. Glista D. Seelisch A. (2013). Nonlinear frequency compression effects on sound quality ratings of speech and music. Trends Amplif, 17, 54–68. 23539261 10.1177/1084713813480856 PMC4040861

[R43] Picou E. M. (2020). MarkeTrak 10 (MT10) Survey Results Demonstrate High Satisfaction with and Benefits from Hearing Aids. Semin Hear, 41, 021–036. 10.1055/s-0040-1701243 PMC7010487 32047346

[R44] Picou E. M. Davis H. Lewis D. Tharpe A. M. (2020a). Contralateral routing of signals systems can improve speech recognition and comprehension in dynamic classrooms. J Speech Lang Hear Res., 63, 2468–2482. 32574079 10.1044/2020_JSLHR-19-00411

[R45] Picou E. M. Lewis D. Angley G. Tharpe A. M. (2020b). Rerouting hearing aid systems for overcoming limited useable unilateral hearing in dynamic classrooms. Ear Hear, 41, 790–803. 31584502 10.1097/AUD.0000000000000800 PMC7117994

[R46] Pittman A. (2011a). Age-related benefits of digital noise reduction for short-term word learning in children with hearing loss. J Speech Lang Hear Res., 54, 1448–1463. 21646423 10.1044/1092-4388(2011/10-0341)

[R47] Pittman A. (2011b). Children’s performance in complex listening conditions: Effects of hearing loss and digital noise reduction. J Speech Lang Hear Res., 54, 1224–1239. 21330646 10.1044/1092-4388(2010/10-0225)

[R48] Pittman A. L., & Hiipakka M. M. (2013). Hearing impaired children’s preference for, and performance with, four combinations of directional microphone and digital noise reduction technology. J Am Acad Audiol, 24, 832–844. 24224990 10.3766/jaaa.24.9.7

[R49] R Core Team. (2023). R: A language and environment for statistical computing.

[R50] Ricketts T. A., & Hornsby B. W. (2003). Distance and reverberation effects on directional benefit. Ear Hear, 24, 472–484. 14663347 10.1097/01.AUD.0000100202.00312.02

[R51] Ricketts T. A., & Hornsby B. W. (2006). Directional hearing aid benefit in listeners with severe hearing loss. Int J Audiol, 45, 190–197. 16579494 10.1080/14992020500258602

[R52] Ricketts T. A., & Picou E. M. (2013). Speech recognition for bilaterally asymmetric and symmetric hearing aid microphone modes in simulated classroom environments. Ear Hear, 34, 601–609. 23524508 10.1097/AUD.0b013e3182886d1e

[R53] Ricketts T. Galster J. Tharpe A. M. (2007). Directional benefit in simulated classroom environments. Am J Audiol, 16, 130–144. 18056881 10.1044/1059-0889(2007/017)

[R67] Ricketts T. (2000). Directivity quantification in hearing aids: Fitting and measurement effects. Ear Hear, 21, 45-58. 10708073 10.1097/00003446-200002000-00008

[R54] Ricketts T. A. Picou E. M. Galster J. (2017). Directional microphone hearing aids in school environments: Working toward optimization. J Speech Lang Hear Res, 60, 263–275. 28114614 10.1044/2016_JSLHR-H-16-0097

[R55] Ricketts T. A. Bentler R. Mueller G. H. (2019). Essentials of Modern Hearing Aids: Selection, Fitting, and Verification. Plural Publishing.

[R56] Scollie S. (2007, June 20, 2016). DSL version v5.0: Description and Early Results in Children Retrieved June 22, 2016, http://www.audiologyonline.com/articles/dsl-version-v5-0-description-959

[R57] Scollie S. Levy C. Pourmand N. Abbasalipour P. Bagatto M. Richert F. Moodie S. Crukley J. Parsa V. (2016). Fitting noise management signal processing applying the American Academy of Audiology pediatric amplification guideline: Verification protocols. J Am Acad Audiol, 27, 237–251. 26967364 10.3766/jaaa.15060

[R58] Shield B., & Dockrell J. E. (2004). External and internal noise surveys of London primary schools. J Acoust Soc Am, 115, 730–738. 15000185 10.1121/1.1635837

[R59] Spratford M. Walker E. A. McCreery R. W. (2019). Use of an application to verify classroom acoustic recommendations for children who are hard of hearing in a general education setting. Am J Audiol, 28, 927–934. 31682768 10.1044/2019_AJA-19-0041 PMC7210436

[R60] Standing L., & Stace G. (1980). The effects of environmental noise on anxiety level. J Gen Psychol, 103, 263–272. 7441223 10.1080/00221309.1980.9921007

[R61] Stelmachowicz P. Lewis D. Hoover B. Nishi K. McCreery R. Woods W. (2010). Effects of digital noise reduction on speech perception for children with hearing loss. Ear Hear, 31, 345–355. 20081536 10.1097/AUD.0b013e3181cda9ce PMC2864336

[R62] Valente D. L. Plevinsky H. M. Franco J. M. Heinrichs-Graham E. C. Lewis D. E. (2012). Experimental investigation of the effects of the acoustical conditions in a simulated classroom on speech recognition and learning in children. J Acoust Soc Am, 131, 232–246. 22280587 10.1121/1.3662059 PMC3283898

[R63] Wolfe J. Duke M. Schafer E. Jones C. Rakita L. (2017). Evaluation of adaptive noise management technologies for school-age children with hearing loss. J Am Acad Audiol, 28, 415–435. 28534732 10.3766/jaaa.16015

[R64] Wolfe J. Duke M. Miller S. Schafer E. Jones C. Rakita L. Dunn A. Browning S. Neumann S. (2022). Evaluation of potential benefits and limitations of noise-management technologies for children with hearing aids. J Am Acad Audiol, 33, 66–74. 35512843 10.1055/s-0041-1735802

[R65] Wu Y. -H. Stangl E. Chipara O. Hasan S. S. DeVries S. Oleson J. (2019). Efficacy and effectiveness of advanced hearing aid directional and noise reduction technologies for older adults with mild to moderate hearing loss. Ear Hear, 40, 805–822. 30379683 10.1097/AUD.0000000000000672 PMC6491270

[R66] Živojinović J. I. Soldatović I. Backović D. Vukašinović D. Babić S. Tomanić M. Ilić B. Vlaisavljević Ž (2023). Personal listening device use and attitude to noise in relation to depression and anxiety among medical students. Noise Health, 25, 176–182. 37815079 10.4103/nah.nah_27_23 PMC10747810

