# Supplementary material for: Do Adolescent Hearing Aid Users Prefer Digital Noise Reduction to Be Activated? Findings From the Laboratory and Home Environments
Source: Ear Hear. 2026 Feb 13;47(4):992–1002. doi: 10.1097/AUD.0000000000001794 (PMC13252974; doi:10.1097/AUD.0000000000001794)
Supplement: Supplementary file 1 [file aud-47-0992-s001.pdf]

Supplemental Digital Content 1

Summary of test box measures

To evaluate the electroacoustic consequences of the advanced digital noise reduction, several test box measures were conducted, all in the Verifit 2 (AudioScan) with a 0.6 cc coupler, a medium receiver, and a hearing aid that was programmed with the mean audiometric thresholds of study participants (displayed in Figure 1 of the main manuscript). The test box measures were repeated with the advanced digital noise reduction settings, using the Dynamic Noise Cancellation control in the Target Phonak programming software, to be either off (0), weak (8), or strong (20).The hearing aid was in a "Speech in Noise" program with the directional microphone set to "Ultrazoom" for all testing.

To ensure the advanced digital noise reduction did not modify gain for speech in quiet, Speech Mapping was conducted using recorded speech (i.e., carrot passage) with a 65 dB SPL input. The results revealed that hearing aid gain in all settings was identical (differences < 1 dB across all frequencies). To evaluate differences in noise reduction for steady state signals from the front, the 'noise reduction' module of the Verifit was used with several different stimulus types (vacuum cleaner, pink noise). None of the signals revealed differences between advanced digital noise reduction settings, which were identical. All three settings resulted in noise reduction estimates of 3, according to the Verifit noise reduction module. These results are consistent with the function of the advanced noise reduction algorithm, which works in combination with a directional beamformer to improve the signal-to-noise ratio in challenging situations. Such an algorithm would not be expected to affect speech in quiet or signals from the front.

Therefore, to evaluate the advanced digital noise reduction algorithm in a manner consistent with its function, the directional microphone module of the Verifit was used (75 dB speech with a 0 dB signal-to-noise ratio). The measurement was completed three times. The results, displayed in Figure S1.1, demonstrate that for signals from the front, all three settings result in similar hearing aid output. For signals from the back, the 'off' setting has a reduced hearing aid output, relative to the front speaker, because even in the 'off' setting, the beamformer is active. With the advanced noise reduction algorithm set to 'weak,' the hearing aid output is reduced relative to the 'off' condition. Similarly, the 'strong' setting results in an even more reduced output relative to the 'off' setting and reduced output relative to the 'weak' setting for signals from the back. Combined, these findings demonstrate that the advanced noise reduction algorithm is affecting hearing aid response for signals from the back, reducing hearing aid output even more than with the directional beamformer alone. The advanced noise reduction algorithm does not appear to be affecting speech in quiet or signals from the front.

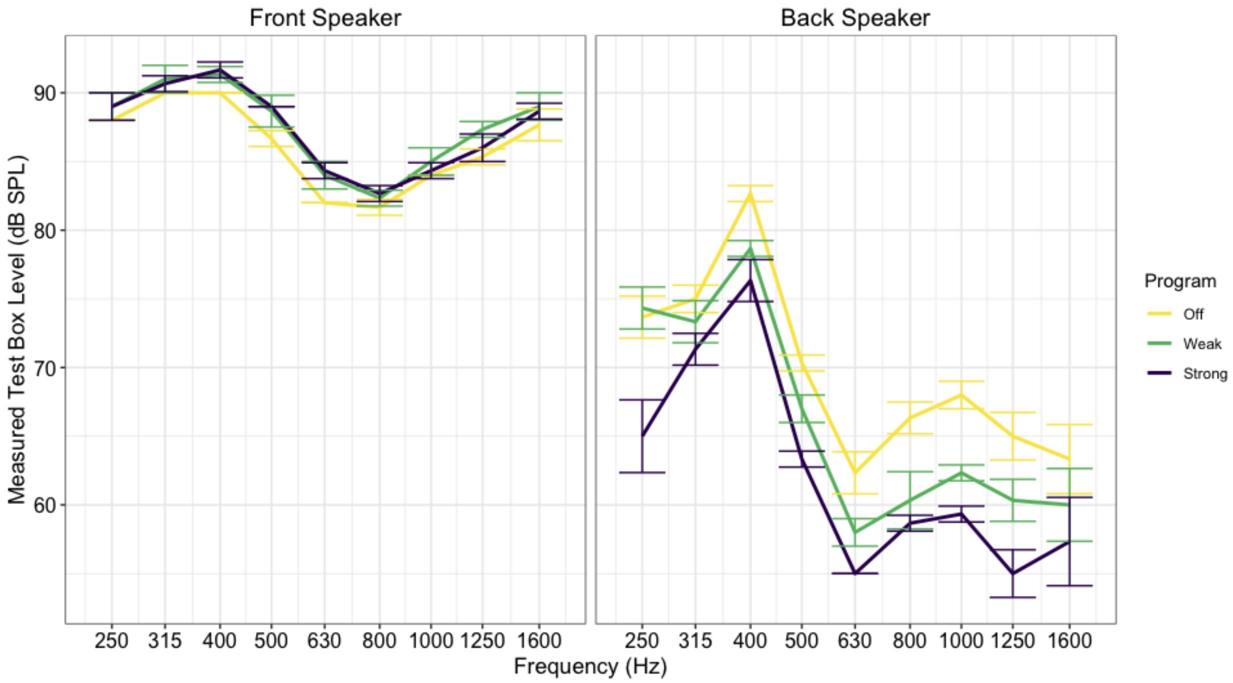

Figure S1.1 Measured hearing aid output in a Verifit 2 test box with three levels of advanced digital noise reduction algorithm (off, weak, strong). The hearing aids were programmed for the mean audiometric thresholds of study participants. Error bars represent +/- 1 standard deviation from the mean across three test measurements. The test signals were speech and noise at a 0 dB signal-to-noise ratio with an overall level of 75 dB SPL.
