# Supplementary figures and images for: Do Adolescent Hearing Aid Users Prefer Digital Noise Reduction to Be Activated? Findings From the Laboratory and Home Environments
Source: Ear Hear. 2026 Feb 13;47(4):992–1002. doi: 10.1097/AUD.0000000000001794 (PMC13252974; doi:10.1097/AUD.0000000000001794)

Left

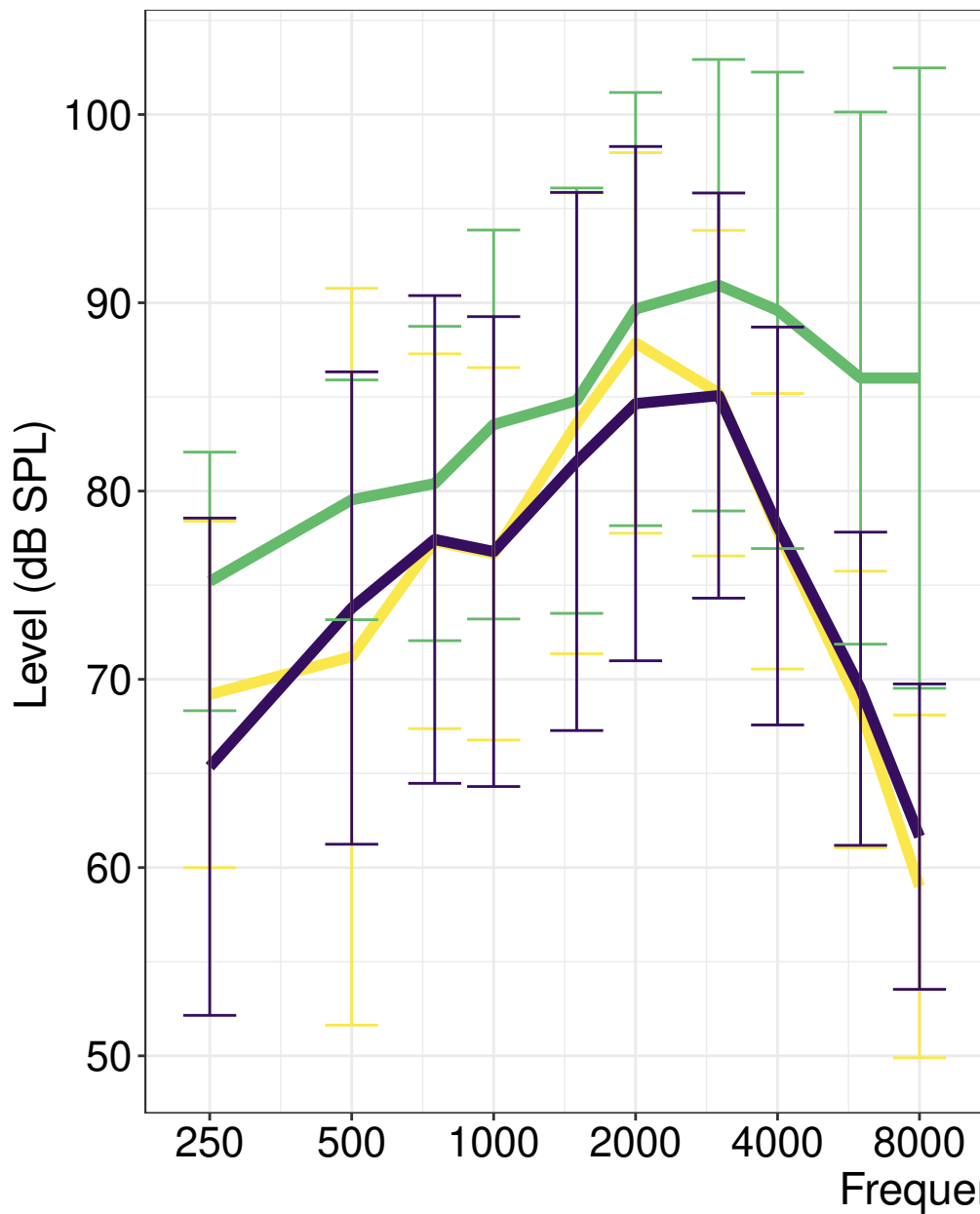

Right

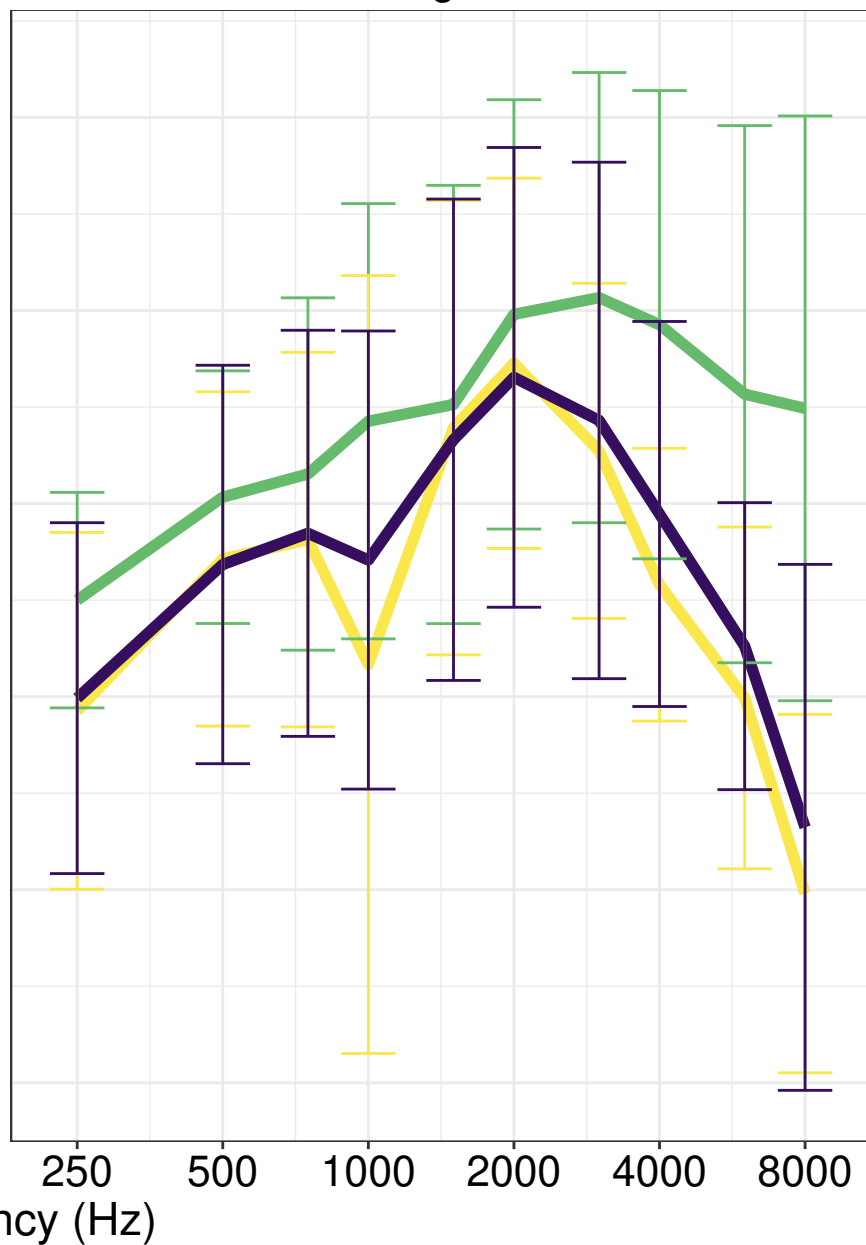

Prescriptive Target    Personal Hearing Aids    Research Hearing Aids

Supplement: Supplementary file 2 [file aud-47-0992-s002.pdf]
