# Supplementary material for: Do Adolescent Hearing Aid Users Prefer Digital Noise Reduction to Be Activated? Findings From the Laboratory and Home Environments
Source: Ear Hear. 2026 Feb 13;47(4):992–1002. doi: 10.1097/AUD.0000000000001794 (PMC13252974; doi:10.1097/AUD.0000000000001794)
Supplement: Supplementary file 3 [file aud-47-0992-s003.pdf]

Supplemental Digital Content 3

Bespoke questionnaire related to smartphone application use

Did you use the 'Speech Focus' slider on the app during your home trial?

- *I used it a lot (many times a day)*
- *I used it a little (once a day)*
- *I used it sometimes (several times a week)*
- *I used it once or twice*
- *I never used it*

Please explain your answer:

Do you think you would use the 'Speech Focus' slider on the app in the future?

- *I would use it all the time (many times a day)*
- *I would use it sometimes (once a day)*
- *I would use it occasionally (several times a week)*
- *I would use it rarely (Several times a month)*
- *I would never use it*

Please explain your answer:

Do you like being able to change 'Speech Focus' with this app?

- *I liked it a lot*
- *I liked it okay*
- *I didn't like or not like it*
- *I didn't like it much*
- *I really didn't like it*

Please explain your answer:

Did being able to adjust the sound with the 'Speech Focus' help you feel more confident in noisy situations?

- *I felt a lot more confident*
- *I felt a little more confident*
- *It didn't change my confidence*
- *It made me feel a little less confident*
- *It made me feel a lot less confident*

Please explain your answer:

Do noisy situations make you feel anxious?

- *I feel very anxious in noise*
- *I feel a little anxious in noise*
- *I don't feel anxious or calm in noise*
- *I feel a little calm in noise*

- *I feel very calm in noise*

Please explain your answer:

Did using 'Speech Focus' change the way you feel in noise?

- *I felt a lot less anxious in noise*
- *I felt a little less anxious in noise*
- *It didn't change the way I feel*
- *I felt a little more anxious in noise*
- *I felt a lot more anxious in noise*

Please explain your answer:
